# A Single-Center Follow-Up Study of Low-Grade Gastric Intraepithelial Neoplasia and the Screening of Key Genes of Precancerous Lesions

**DOI:** 10.3389/fonc.2022.899055

**Published:** 2022-06-30

**Authors:** Xiao-Xu Jin, Xiao-Li Xie, Fu Niu, Kai-Ge Yin, Chen-Guang Ji, Jin-Feng Cui, Li Liu, Zhi-Jie Feng

**Affiliations:** ^1^ Department of Gastroenterology, The Second Hospital of Hebei Medical University, Hebei Key Laboratory of Gastroenterology, Hebei Institute of Gastroenterology, Hebei Clinical Research Center for Digestive Diseases, Shijiazhuang, China; ^2^ Department of Palliative Treatment, The Eighth People’s Hospital of Hebei Province, Shijiazhuang, China

**Keywords:** follow-up study, key genes, database, gastric cancer, risk factors

## Abstract

**Objective:**

The study aimed to summarize the morphological characteristics of low-grade gastric intraepithelial neoplasia (LGIN) and explore its outcomes and risk factors. Additionally, it aimed to screen the core different expression genes (DEGs) of high-grade gastric intraepithelial neoplasia (HGIN) using bioinformatics methods to identify biomarkers for early gastric cancer outcomes.

**Methods:**

The clinical and pathological data of 449 patients with LGIN in the endoscopy center of the Second Hospital of Hebei Medical University from June 2013 to September 2018 were collected for retrospective analysis. The GSE130823 and GSE55696 data sets were selected from the Gene Expression Omnibus database, and the GEO2R tool was used to screen DEGs in HGIN and chronic gastritis tissue types. A DEG functional enrichment analysis was conducted using the Database for Annotation, Visualization, and Integrated Discovery. The STRING database was utilized to create a protein–protein interaction network, and the CytoHubba plug-in was used to screen the key genes of HGIN.

**Results:**

The incidence of LGIN increased with age, and most of the patients were aged between 45–59 years (P = 0.048). Lesions were found mainly in the cardia, mostly in people aged 60 (P < 0.05). Progression occurred in 42 of 449 patients, with a 9.4% rate of cancer development. Foci larger than 10 mm, ulcerative lesions, and an Helicobacter pylori-positive result were factors affecting the outcome of LGIN (P < 0.05). Seven core genes of HGIN were screened, including MYC, SOX2, CDX2, TBX3, KRT7, CDKN2A, and MUC5AC.

**Conclusion:**

The patients with LGIN reflected the potential for developing cancer. A magnifying gastroscope can contribute to the detection of early gastric cancer. Additionally, the MYC, CDX2, and TBX3 genes may act as specific biomarkers of HGIN.

## Introduction

In China, gastric cancer is the second-most common cancer and the third leading cause of tumor-related death (1, 2). Early detection, diagnosis, and treatment are the main strategies for improving prognosis and reducing the mortality rate in gastric cancer cases. Correa et al. (3) presented the occurrence of gastric cancer as a multi-stage and multi-factor process. The recognized development mode of gastric cancer is as follows: chronic atrophic gastritis → intestinal metaplasia → intraepithelial neoplasia → early gastric cancer. Precancerous lesions are closely related to the occurrence of gastric cancer. Gastric intraepithelial neoplasia (GIN) is an important precancerous lesion that is classified as low-grade GIN (LGIN) and high-grade GIN (HGIN); these terms have replaced previous ones, such as dysplasia, atypical hyperplasia, and carcinoma *in situ*. Low-grade GIN corresponds to mild and moderate gastric dysplasia, while HGIN corresponds to severe gastric dysplasia and carcinoma *in situ*. Studies (4) have shown that HGIN has a high rate of cancer development, and in China, endoscopic resection and regular follow-ups are recommended as an approach to treatment.

The natural history of GIN has not been clarified. Accordingly, the natural process of LGIN remains poorly understood. A few lesions may progress to invasive carcinoma (5); accordingly, it is recommended that regular endoscopic examinations be performed and the risk of progression into cancer is monitored. In this study, the clinical and pathological data of 449 patients who were pathologically confirmed with LGIN in the Endoscopy Center of the Second Hospital of Hebei Medical University from June 2013 to September 2018 were collected for follow-up and retrospective analysis to guide clinical practices.

The molecular mechanism of gastric cancer is still unclear, and there is a lack of clinical biomarkers for its early diagnosis. In recent years, the high-throughput technique has been widely used in the context of many diseases (6) and has demonstrated significant advantages for screening disease-related mutation genes, finding new therapeutic targets, and providing personalized treatment, which has strongly promoted the progression of disease genomics. The current study screened the different expression genes (DEGs) of HGIN and gastritis using the GEO database and screened core genes using Gene Ontology (GO) functional annotation, the Kyoto Encyclopedia of Genes and Genomes’ (KEGG) enrichment pathway analysis, and a protein–protein interaction network (PPI) to provide new ideas for the precise diagnosis of precancerous lesions.

## Materials and Method

### General Information

#### Clinical Data

This study collected the clinical data, endoscopic morphologies, and histological cresyl violet acetate staining information related to the biopsy specimens of 449 patients who were pathologically confirmed with LGIN in the Endoscopy Center of the Second Hospital of Hebei Medical University from June 2013 to September 2018. These patients were aged between 22 and 80 years and included 270 males and 179 females.

The inclusion criteria were as follows: ① Patients who agreed to this diagnosis and its observation and whose gastroscopic pathological findings indicated LGIN, ② patients who were conscious, had an informed understanding of the present study, and voluntarily provided informed signed consent for participation in the research, and ③ patients who accepted related data statistics during the experiment.

The exclusion criteria were as follows: ① Patients with HGIN and severe dysplasia, ② patients with a past medical history of gastrointestinal tumors and a history of surgery and/or chemotherapy, ③ patients with distant metastasis, ④ patients with severe mental disorders, cognitive disorders, or multiple organ dysfunction, and ⑤ patients with contraindications for endoscopic examination (7).

The termination criteria were as follows: ① Serious adverse events, such as serious complications resulting from endoscopic procedures, ② a follow-up time shorter than 3 years, ③ a missed follow-up, and ④ active withdrawal from the study.

#### Microarray Data

The key phrase “early gastric cancer” was used as a search term in the GEO database (https://www.ncbi.nlm.nih.gov/geo/); as a result, two microarray data sets, i.e., GSE130823 (8) and GSE55696 (9) were identified that simultaneously provided gene chips from gastritis, LGIN, HGIN, and early gastric cancer tissue types. The GSE130823 data set was based on the GPL17077 platform [Agilent-039494 SurePrint G3 Human GE v.2 8 × 60 K Microarray 039381 (Probe Name version)], which comprised 94 samples, including 47 gastritis, 17 LGIN, 14 HGIN, and 16 early gastric cancer samples. The GSE55696 data set was based on the GPL6480 platform [Agilent-014850 Whole Human Genome Microarray 4 × 44 K G4112F (Probe Name version)], which comprised 77 specimens, including 19 gastritis, 19 LGIN, 20 HGIN, and 19 early gastric cancer samples. All data are accessible online free of charge. Information related to the data sets is shown in **Table 1**.

**Table 1:** Details for GEO early gastric cancer data.

Reference	Sample	GEO	Platform	HGIN	Gastritis
Zhang et al. (2020) (8)	Stomach	GSE130823	GPL17077	14	47
Xu et al. (2014) (9)	Stomach	GSE55696	GPL6480	20	19

GEO, Gene Expression Omnibus; HGIN, High Grade Intraepithelial Neoplasia.

### Method

#### Study Methods for Clinical Data

Follow-up observations were arranged at the center for 449 patients with LGIN. These patients were given oral pronase to remove gastric mucus 20 minutes before examination. A gastroscopy was performed on the patients using the following Olympus and Fujifilm instruments: an Olympus endoscope host system (CV-260, CV-290, Olympus, Japan) and gastroscope (GIF-Q260J, GIF-HQ290, Olympus, Japan), a Fujifilm endoscope host system (VP-4450HD, Fujifilm, Japan) and gastroscope (EG-590WR, Fujifilm, Japan), and disposable coated endoscopic biopsy forceps (MTN-BF-23/18-A-C-2, MICRO-TECH [Nanjing] Co., Ltd., China). The morphology of the patients’ microglandular tubes and microvessels could be observed clearly to guide the biopsies when narrow-band imaging or flexible spectral imaging color-enhancement models were used. According to the Paris (10) classification criteria, the endoscopic morphology of GIN was divided into protruded (type I), superficial (type II), and depressed (type III) types. According to the Vienna classification of gastrointestinal epithelial neoplasia (11), biopsy specimens were pathologically confirmed with LGIN *via* biopsy. A regular follow-up of the lesions was recommended, and the initial biopsy results were used as raw data. At least four samples from the same sites were taken for biopsy during patients’ return visits. The specimens were histologically stained with cresyl violet acetate to determine the presence of Helicobacter pylori. The final examination was taken as representing the final follow-up results. The follow-up period was 5 years, with an interval of 6–12 months. If there were any signs of dysplasia or histological malignancy, patients were treated with endoscopic submucosal dissection (ESD) or surgery. All patients provided informed written consent prior to undergoing surgery.

#### Bioinformatics Analysis Methods

##### Screening Different Expression Genes

The GEO (12) database archives many high-throughput functional genome studies that include data that has been processed and normalized using various methods. After data normalization, a principal-component analysis of the LGIN and HGIN tissue types in the patients revealed unsatisfactorily discrete conditions. This may have been due to insignificant differences or differences in the pathological diagnoses of LGIN and HGIN. Therefore, tissue specimens of HGIN and gastritis were selected for difference analysis. The GEO2R (http://www.ncbi.nlm.nih.gov/geo/geo2r/) interactive network tool was used to screen the DEGs of HGIN and gastritis tissues. The adjusted P < 0.05 and |logFC| > 1 values were used as screening indices, and the Benjamini–Hochberg procedure was used by default to control the false discovery rate. Venn diagrams (http://bioinformatics.psb.ugent.be/webtools/venn/) were used to determine the overlapped DEGs of the GSE130823 and GSE55696 data sets.

##### Functional and Pathway Enrichment Analysis

The Database for Annotation, Visualization and Integrated Discovery (DAVID) (13) (http://david.abcc.ncifcrf.gov/) is an online database that integrates annotation, visualization, and integrated discovery that can be used to conduct enrichment analyses on the functions and pathways of candidate encoded proteins. The database provides researchers with a set of comprehensive functional annotations to better understand the biological significance of genes. Gene Ontology and KEGG enrichment analyses were performed for the identified DEGs using DAVID, and the “stringi,” “ggplot2,” and “dplyr” packages of the R programming language were used to output the results visually. This study analyzed the significantly up-regulated and down-regulated DEGs that were determined from the integrated microarray HGIN data. The value of P < 0.05 was considered statistically significant.

##### Establishing the Protein–Protein Interaction

The STRING database (v.11.5) is online software that is typically used to identify the interaction between known and predicted proteins. The results in the database are sourced mainly from experimental data, databases, data mining, and predicted bioinformatics data. In addition, the core of Cytoscape software is network. Each node represents a gene, protein, or molecule, and the connections between the nodes represent the interactions between these biomolecules. To assess the interaction between DEGs, they were mapped to the STRING database. Only an overall score of >0.4 was defined as significant. Cytoscape software was used to create a PPI network, and the software’s CytoHubba plug-in was used to screen the top-10 core genes according to the degree of gene nodes.

##### Initial Verification

The GSE55696 data set was used, and the data were downloaded in the MINiML format. The expression levels of the screened core genes were analyzed in the inflammation (n = 19) and HGIN (n = 20) groups. After data normalization, the results were achieved using the box plot of the “ggplot2” function in the R package.

#### Statistical Analysis

A statistical analysis was carried out using SPSS Statistics 17.0 software. A t-test and a continuity correction test were used for comparison between the groups; P < 0.05 was considered to reflect a statistically significant difference, and the test level was α = 0.05. A Wilcoxon rank–sum test was used to determine the differences related to the genetic structure of different tissue. A bioinformatics analysis was performed using GEO2R, the DAVID database, Cytoscape and CytoHubba software, and the related R software (v.4.1.2).

## Results

### Statistical Results of the Clinical Data

#### Endoscopic Findings of Low-Grade Gastric Intraepithelial Neoplasia

Protruded (type I) lesions was identified in 6 patients, including 3 patients with Is and 3 patients with Ip. Superficial (type II) lesions was found in 439 patients, including 201 patients with IIa, 15 patients with IIb, and 223 patients with IIc. Ulcerative (type III) lesions was found in 4 patients.

#### Association Between Lesion Position and Age in Low-Grade Gastric Intraepithelial Neoplasia

Of the 449 patients with LGIN, 201 were aged 45–59 years, accounting for 44.8%. The lesions, which occurred mainly in the cardia, were found primarily in people aged 60, accounting for 58.1%. The differences between the 45–59 age group and other age groups were statistically significant (**χ** ^2^ = 13.21, P = 0.048). There was no statistically significant difference in LGIN between other positions except for cardia (**χ** ^2^ = 0.97, P = 0.660). The association between the lesion position and the age of the patients with LGIN is shown in **Table 2**.

**Table 2:** Association between lesion position and age of patients with low gastric intraepithelial neoplasia.

Position	n	Age			
		<30	30-44	45-59	≥60
Gastric antrum	272	7 (2.6)	34 (12.5)	132 (48.5)	99 (36.4)
Cardia^*^	62	0 (0.0)	6 (9.7)	20 (32.3)	36 (58.1)
Gastric body	33	2 (6.1)	2 (6.1)	14 (42.4)	15 (45.5)
Pylorus	16	2 (12.5)	3 (18.8)	6 (37.5)	5 (31.3)
Gastric angle	66	4 (6.1)	10 (15.2)	29 (43.9)	23 (34.8)
Total	449	15 (3.3)	55 (12.2)	201 (44.8)	178 (39.6)

^*^P < 0.05.

#### Outcome and Evaluation of Related Indices of Low-Grade Gastric Intraepithelial Neoplasia in Different Groups

**Figure 1:**
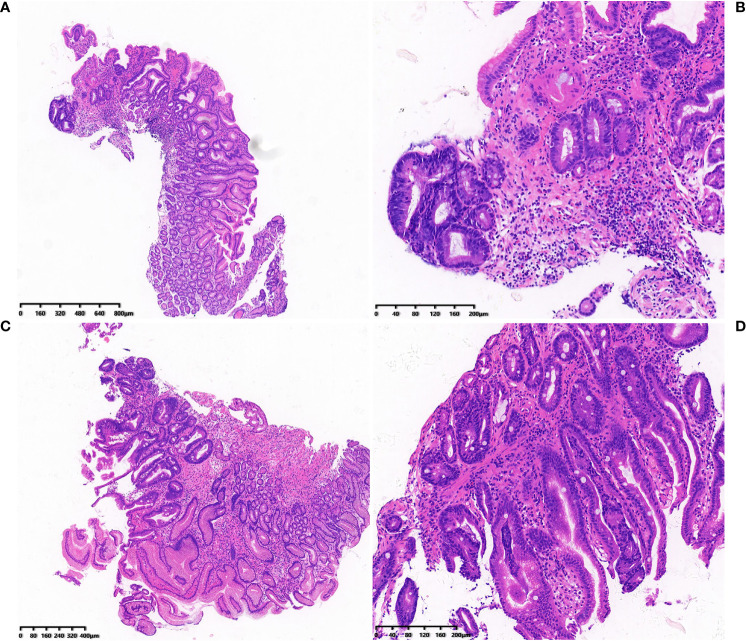
**(A-D)** Histological pictures of the cases with low grade gastric intraepithelial neoplasia who developed cancer.

Based on the pathological results of a follow-up biopsy, 449 patients with LGIN were divided into two groups (stable and progression groups). The stable disease group included improved/unchanged lesions. The disease progression group included progression to HGIN or cancer development. The lesion size was measured by comparing the size of the lesion with biopsy forceps. In each gastroscopy, at least four biopsy specimens were taken from the same GIN tissue that had been examined. The biopsy specimens were fixed immediately in an 8% formaldehyde solution and sent to the Department of Pathology to determine the presence of H. pylori by histological cresyl violet acetate staining. In this study, the progression group included 42 patients with a 9.4% rate of cancer development (42/449). Among them, 34 patients developed HGIN, and 8 patients (3 males and 5 females) developed cancer, including 2 patients with signet-ring cell carcinoma and 6 patients with adenocarcinoma. The range of lesions larger than 10 mm, ulcerative lesions, and those that were Hp positive were risk factors affecting the outcome of LGIN (P < 0.05). There was no statistical difference in gender between the groups (**χ** ^2^ = 0.558, P = 0.455), although there were statistical differences in Hp (**χ** ^2^ = 38.76, P = 0.00), surface morphology (**χ** ^2^ = 12.286, P = 0.006), and lesion size (**χ** ^2^ = 7.241, P = 0.007) between the groups. The outcome of LGIN in the stable and progression groups is shown in **Table 3**. Some histological pictures of the cases with low grade gastric intraepithelial neoplasia who developed cancer are shown in **Figure 1**.

**Table 3:** Outcome of low gastric intraepithelial neoplasia in stable and progression groups.

Item	Stable disease group	Disease progression group	χ^2^ value	P value
**Gender**				
Male	247	23	0.558^a^	0.455
Female	160	19		
**Lesion size**				
≤10mm	398	38	7.241^a^	^*^0.007
>10mm	9	4		
**Hp**				
Positive	345	19	38.761^a^	^*^<0.001
Negative	62	23		
**Surface morphology**				
Congestion	45	1	12.286^a^	^*^0.006
Ulcer	6	0		
Erosion	216	34		
Flat	139	7		

a Chi-squared test with continuity correction; ^*^P < 0.05.

### Statistical Results of the Bioinformatics Data

#### Identification of Different Expression Genes

**Figure 2:**
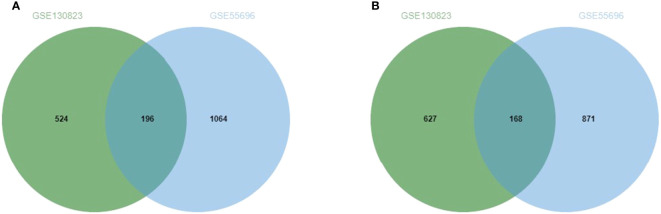
**(A)** Up-regulated genes and the intersection part of the two circles is the number of DEGs intersection of dataset GSE130823 and GSE55696. **(B)**Down-regulated genes and the intersection part of the two circles is the number of DEGs intersection of dataset GSE130823 and GSE55696.

The GEO2R online analysis tool was used to screen out the DEGs of two microarray data sets (GSE130823 and GSE55696). According to the adjusted standards of P < 0.05 and |log FC|≥1.0, 1,151 DEGs were screened from GSE130823, including 524 up-regulated expression genes and 627 down-regulated expression genes. A total of 1,935 DEGs were screened from GSE55696, including 1,064 up-regulated expression genes and 871 down-regulated expression genes. A total of 364 DEGs were screened from the two data sets, including 196 genes with up-regulated expression in HGIN tissue and 168 genes with down-regulated expression in HGIN tissue. Overlapped DEGs from the GSE130823 and GSE55696 data sets were obtained *via* the analysis of Venn diagrams (http://jvenn.toulouse.inra.fr/app/example.html) (see **Figure 2**).

#### Gene Ontology Function and Kyoto Encyclopedia of Genes and Genomes Pathway Enrichment Analyses of Different Expression Genes

**Figure 3:**
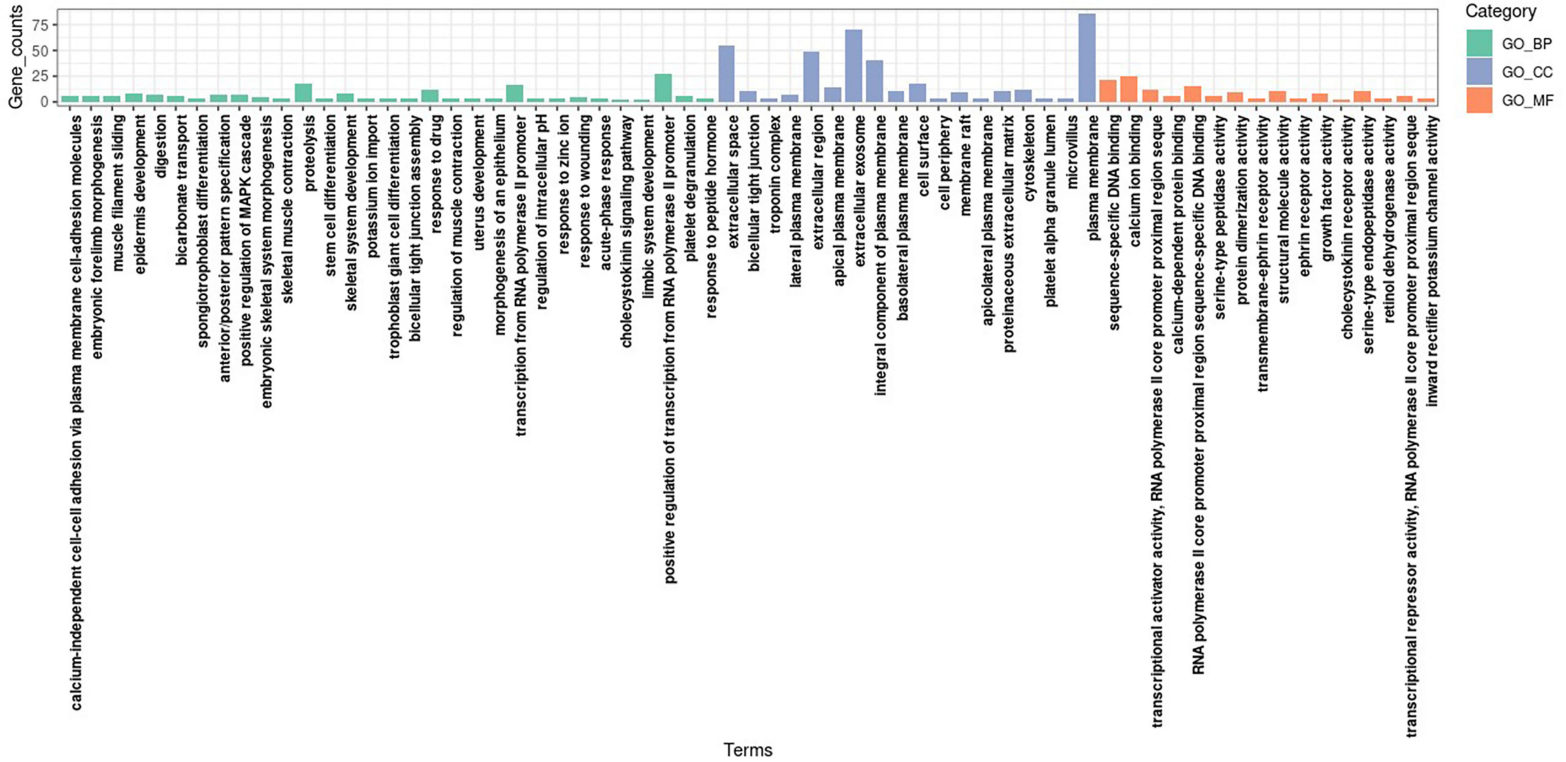
GO enrichment analysis results of DEGs.

**Figure 4:**
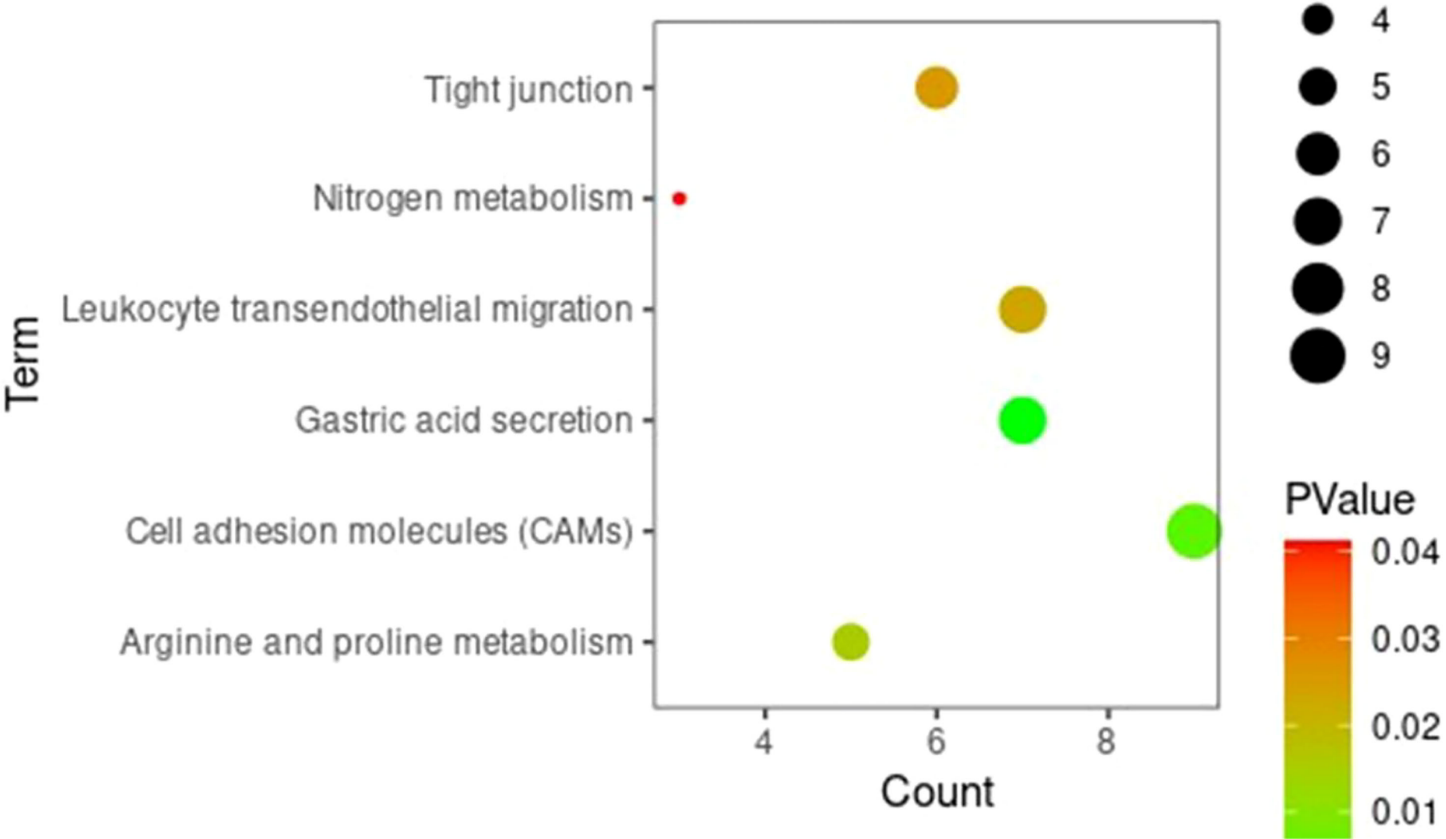
KEGG signaling pathway analysis results of DEGs.

Gene ontology and KEGG enrichment analyses were conducted for DEGs to clarify the biological processes, cellular components, molecular functions, and signaling pathways in which marker genes may be involved. The GO analysis showed that, in terms of biological processes, DEGs were involved mainly in the regulation of cell proliferation, the positive regulation of the transcription of ribonucleic acid (RNA) polymerase II promoters, digestion, and the positive regulation of mitogen-activated protein kinase cascades. In terms of cell components, DEGs were mainly distributed in extracellular domains, extracellular exosomes, and plasma membranes. In terms of molecular function, DEGs were involved mainly in sequence-specific deoxyribonucleic acid (DNA) binding, calcium ion binding, and RNA polymerase II core promoter proximal region sequence-specific DNA binding. The KEGG signaling pathway analysis showed that DEGs were involved mainly in gastric acid secretion, the transendothelial migration of white blood cells, protein metabolism, CAMs, and other pathways. **Figures 3** and **4** present the results of the analyses, respectively.

#### Creation of Protein–Protein Interaction and Core Genetic Screening

**Figure 5:**
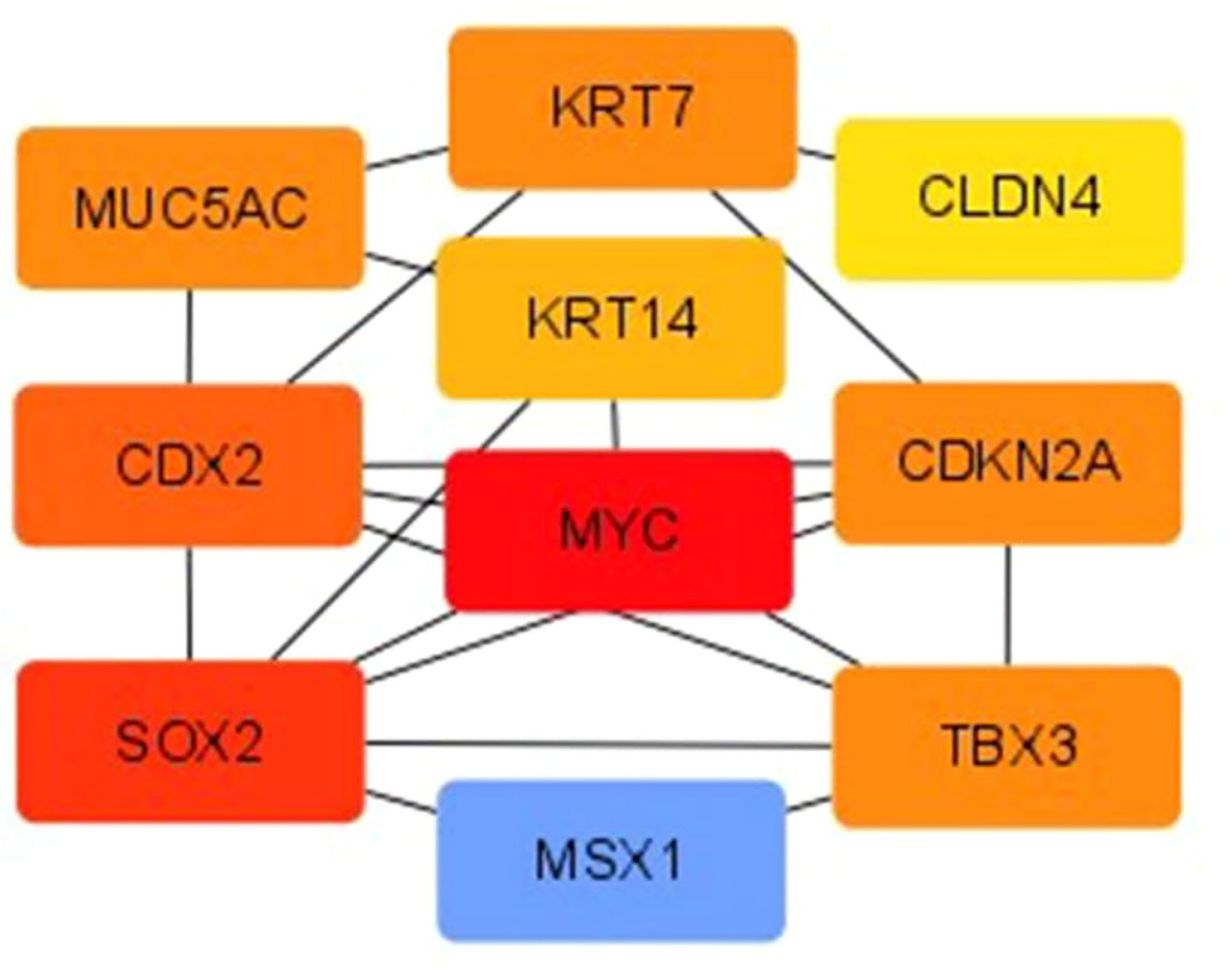
PPI diagram of DEGs.

The PPI of DEGs was established using the STRING database, and PPI images were output visually using Cytoscape. The top-seven core genes were screened according to the degree of gene nodes using Cytoscape’s CytoHubba plug-in, as follows: MYC (62 scores), SOX2 (46 scores), Caudal-type homeobox 2 (CDX2) (34 scores), TBX3 (22 scores), KRT7 (22 scores), CDKN2A (22 scores), and MUC5AC (22 scores) (see **Figure 5**).

#### Expression Level of Core Genes

**Figure 6:**
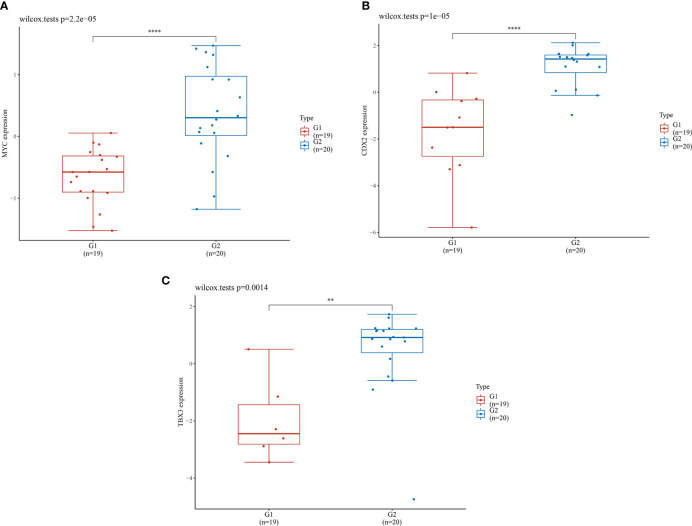
Expression levels of 3 genes in different groups. G1 represents the inflammation group (n=19), G2 represents the HGIN group (n=20). The horizontal axis represents samples in different groups and the vertical axis represents the gene expression distribution. Different colors represent different groups. The left upper corner represents the significance p-value test method. **(A)** Expression distribution of MYC gene in different tissues; **(B)** Expression distribution of CDX2 gene in different tissues; **(C)** Expression distribution of TBX3 gene in different tissues. (**P < 0.01,****P < 0.0001).

The seven core genes screened above were verified in the inflammation and HGIN groups. It was found that the expression levels of MYC, CDX2, and TBX3 in the HGIN group were significantly elevated (P < 0.01), as shown in **Figure 6**.

## Discussion

Gastric intraepithelial neoplasia is regarded as a precancerous lesion; therefore, its early diagnosis and prevention are important. It is the focus of gastric cancer prevention and treatment, and there is a lot of space for reversal. The outcome of LGIN is rarely reported at home or abroad, and statistical data differ across regions. Our study shows that the disease is most common in the 45–59 age group in this region(Hebei province, China), which coincides with the recommendation of “China’s Consensus on Early Cancer” (14) that the age for initial gastric cancer screening is lowered to 40 years. The rate of LGIN progression to HGIN and invasive carcinoma is high in the region. Statistics from the National Cancer Center show that the incidence and mortality of esophageal cancer in Hebei Province are higher than the national averages, while existing studies have demonstrated that a high incidence of esophageal cancer was associated with local eating habits and the quality of drinking water (15). We considered that the high incidence in this statistical analysis may have been related to the eating habits of residents in surrounding areas; additionally, the sample size was insufficient, which may have given rise to errors. Related studies conducted by clinical centers in China (16, 17) show that the gastric antrum is the most common site of disease, followed by the gastric angle, which is partially consistent with the results of the present study. Therefore, these sites should be screened carefully under observation. The other results are different. This may have been due to the small sample size and the short follow-up period of the present study. Therefore, the sample size must be expanded to determine the distribution positions of precancerous lesions.

Currently, the final diagnosis of GIN depends on histopathology. The complex process of a precancerous lesion’s development to gastric cancer and its progression is a result of multiple factors. Several factors contribute significantly to the increased risk of gastric cancer, such as family history, diet, alcohol consumption, smoking, and infection with H. pylori and the Epstein–Barr virus (18); however, the exact pathogenesis remains unclear. Diet, NSAID use and history of smoking are relevant risk factors for LGIN. In the next study, we will integrate such clinical information and perform univariate and multifactorial regression analysis to obtain LGIN-related risk factors and construct an early warning model.

This study revealed that Hp infection was also one of the factors leading to disease progression and the most important risk factor for gastric adenocarcinoma, which causes progressive damage to the gastric mucosa and may eventually lead to atrophic gastritis and subsequent intestinal metaplasia. This is a necessary process of gastric cancer progression (16). High-risk patients with Hp infection should receive normal eradication therapy and change their eating habits. Type II was found to be the most common lesion morphology in the current study. Yu et al. (19) reported that type IIc was the most common in 130 patients with early gastric cancer, redness was the most common color change, and lesion surfaces were primarily irregular. Therefore, the discovery of superficial lesions in endoscopic procedures requires attention. A meta-analysis (20) indicated that a depressed lesion with a diameter of >2 cm was an influencing factor for underestimation in the LGIN group. In this present study, 1 cm was taken as the limit and indicated statistical significance. Healthcare professionals must be vigilant to prevent missed diagnoses when the lesion range is 1 cm.

With the rapid development of endoscopic diagnosis and the high-throughput technique, it is important to identify a molecular marker for the rapid diagnosis of HGIN to increase the detection rate of precancerous lesions. Bioinformatics analyses have been widely used to identify potential biomarkers related to the diagnosis, treatment, and prognosis of gastric cancer (21). Zohreh et al. (15) screened biomarkers related to the early diagnosis of gastric cancer using the bioinformatics weighted gene co-expression network analysis method and verified them using a reverse-transcription quantitative polymerase chain reaction. However, few articles have reported the screening of core genes related to gastric precancerous lesions using bioinformatics methods. The current authors hope to be able to advance the detection of gastric cancer.

In this study, two microarray data sets, i.e., GSE130823 and GSE55696, were integrated, and the GEO2R online analysis tool was used to screen 364 common DEGs, including 196 up-regulated and 168 down-regulated expression genes. Gene Ontology functional annotation indicated that the DEGs of precancerous lesions were found mainly in extracellular exosomes and plasma membranes, which mediated biological functions (such as sequence-specific DNA binding and calcium ion binding) and were involved in the biological processes of cell proliferation, regulation, and digestion. The KEGG enrichment analysis showed that DEGs were involved mainly in the regulation of gastric acid secretion, the transendothelial migration of white blood cells, protein metabolism, and CAMs signaling pathways. The results suggested that these DEGs may play an important role in the progression of precancerous lesions to early gastric cancer. Furthermore, the expression levels of MYC, CDX2, and TBX3 among core genes in the HGIN group were significantly increased, providing a new approach for the diagnosis and treatment of precancerous lesions.

The MYC family of proteins includes c-MYC, n-MYC, and l-MYC. The dysregulation of c-MYC is involved in genome instability, tumor formation, and the maintenance of tumor growth (22). The MYC protein was reported to be a unique carcinogenic driving factor in an article on pan-cancer studies (23), where c-MYC mainly regulated multiple biological processes by selectively activating gene expression. Liu et al. (24) found that the expression levels of c-MYC and PRMT5 were up-regulated in human primary gastric cancer tissues, and the PRMT5-dependent transcriptional repression of c-MYC target genes was necessary for gastric cancer progression, which provided a potential new strategy for the targeted treatment of gastric cancer. However, the expression of MYC in precancerous tissue has not been reported; therefore, its role in this context is unknown.

Caudal-type homeobox 2 plays an important role in the regulation of digestive epithelial mucosa. It can induce gastric intestinal metaplasia and cause abnormal differentiation, eventually leading to cancer development. It was confirmed by immunohistochemistry that the expression level of CDX2 was elevated in patients with early gastric cancer (25). Based on this study, the expression of CDX2 in the tissue of patients with HGIN must be further verified. Chen et al. (26) found that hTERT could up-regulate the expression of CDX2 through the nuclear factor kappa-B signaling pathway to contribute to gastric intestinal metaplasia. Studies have also shown that the over-expression of TBX3 (a member of the T-box transcription-factor family) in human gastric cancer tissue was associated with the advanced stage of tumors and lymph node status and contributes to the growth and invasion of cancer cells (27). As a result, MYC, CDX2, and TBX3 can be used as biomarkers of HGIN.

tIn summary, there are many DEGs present in gastric precancerous tissue, such as MYC, SOX2, CDX2, TBX3, KRT7, CDKN2A, and MUC5AC. Among these, MYC, CDX2, and TBX3 may act as specific biomarkers of HGIN. Additionally, it is recommended that patients with LGIN with the above-noted risk factors undergo core DEG detection in tissue samples to comprehensively assess the risk of HGIN and even the potential presence of early gastric cancer, thereby enhancing the likelihood of positive endoscopic treatment. For example, ESD surgery can benefit patients since it is minimally invasive and typically involves a quick recovery. Patients without risk factors are recommended to have a gastroscopy reexamination every three to six months.

The conclusions of this study require further verification by additional clinical studies. Nonetheless, it provides new ideas for the diagnosis and treatment of patients with precancerous lesions. However, the expression of these DEGs in precancerous tissue has not been experimentally verified. Therefore, the authors aim to perform subsequent immunohistochemistry procedures on patient tissue samples to further verify the conclusions of this study and investigate the potential mechanism of the progression of LGIN to HGIN.

## Conclusion

The LGIN follow-up showed the potential for the progression toward cancer and even its development. This region (Hebei province, China) has a high rate of GIN cancer development. Patients should have an enhanced awareness of the need for gastroscopy screening and re-examination with age, particularly those over the age of 45, to ensure early detection and treatment. Follow-ups with a magnifying endoscope are beneficial for detecting precancerous lesions and preventing further tumor progression. The detection of one case of early cancer means saving a family. The MYC, CDX2, and TBX3 genes were screened using bioinformatics methods to act as specific biomarkers of HGIN.

## Data Availability Statement

The original contributions presented in the study are included in the article/supplementary material. Further inquiries can be directed to the corresponding author.

## Ethics Statement

The studies involving human participants were reviewed and approved by ethics committee of The Second Hospital of Hebei Medical University. The patients/participants provided their written informed consent to participate in this study.

## Author Contributions

Conception and design of the research: X-XJ, X-LX, and Z-JF; Acquisition of data: X-XJ; Analysis and interpretation of the data: K-GY, C-GJ, and J-FC; Statistical analysis: FN and X-XJ; Obtaining financing: Z-JF; Writing of the manuscript: X-XJ; Critical revision of the manuscript for intellectual content: LL and Z-JF. All authors read and approved the final draft.

## Funding

Program of Natural Science Foundation of Hebei: Beijing-Tianjin-Hebei Basic Research Cooperation Special Project (H2018206450). Hebei provincial government funded the project of clinical medical talents (303-2022-27-26).

## Conflict of Interest

The authors declare that the research was conducted in the absence of any commercial or financial relationships that could be construed as a potential conflict of interest.

## Publisher’s Note

All claims expressed in this article are solely those of the authors and do not necessarily represent those of their affiliated organizations, or those of the publisher, the editors and the reviewers. Any product that may be evaluated in this article, or claim that may be made by its manufacturer, is not guaranteed or endorsed by the publisher.
